# Evaluation of the role of silicon in alleviating cadmium stress in sorghum

**DOI:** 10.3389/fpls.2025.1721563

**Published:** 2026-01-05

**Authors:** Hazel Gökdere, Hava Şeyma Inci, Bedriye Bilir

**Affiliations:** 1 Institute of Science, Department of Field Crops, Bingöl University, Bingöl, Türkiye; 2 Department of Crop and Animal Production, Vocational School of Food, Agriculture and Livestock, Bingöl University, Bingöl, Türkiye; 3 Department of Soil Science and Plant Nutrition, Faculty of Agriculture, Kahramanmaraş Sütçü İmam University, Kahramanmaraş, Türkiye

**Keywords:** silicon, toxic metals, sorghum, cadmium, abiotic stress

## Abstract

**Introduction:**

This experiment was designed to examine the mitigating effects of Si treatment on Cd-exposed sorghum plants and was conducted as a pot experiment.

**Methods:**

The experiment was executed using a factorial design in completely randomized design (CRD), including 3 Cd concentrations (0, 10, and 20 mg kg^-1^) and 4 Si concentrations (0, 100, 200, and 300 mg kg^-1^). After 90 days, the plants were harvested, and the Cd, Fe, and Zn concentrations in the plant organs, as well as the morphological characteristics of the plants, were examined.

**Results:**

Cadmium accumulated most in the roots compared to other organs of sorghum; roots>stems>leaves=panicle. Si treatments in Cd_10_ stress reduced Cd concentration in roots and panicles, Fe in stems and panicles, and Zn in roots. Si treatments in Cd_20_ exposure increased Fe in roots, while Zn decreased in Stems and leaves. Si treatments in Cd exposure increased plant stem diameter, dry root weight, and total plant weight. Regarding the role of Si application in alleviating Cd exposure in sorghum, the Cd_10_Si_300_ treatments reduced Cd by 86.6% in panicles, 30.9% in leaves, and 28.9% in stems, while Cd_10_Si_200_ treatments reduced Cd concentration in roots by 38% at most. In Cd translocation to panicles, it was observed that the treatment of 300 mg kg^-1^ Si had a strong potential to inhibit translocation in the presence of about 10 mg kg^-1^ Cd.

**Discussion:**

In the soils with similar properties and Cd contamination used in this study, the treatment of 300 mg kg^-1^ Si is considered to be an important concentration to overcome Cd translocation.

## 1 Introduction

Plants, biodiversity and ecosystems are at risk from a variety of abiotic and biotic stressors, including environmental pollution and global warming. Plants have undergone evolutionary changes over the last 200 years under the influence of increasing abiotic and biotic stresses due to increased human activities (Westwood et al., 2021). Understanding stress tolerance mechanisms in plants has become crucial to develop resilience in plant species to maintain ecosystem, biodiversity and secure food security services (Nawaz et al., 2023).

Cadmium (Cd) is an element that can be found in most environments and is considered one of the hazardous toxic heavy metals (Rai et al., 2016). Cadmium is a non-essential trace element that has a high potential for transfer in soil-plant parts, can be easily transmitted into the food chain and poses a high risk to living organisms (Naz et al., 2025). It is an element that is not necessary for the human body and seriously affects health (Zhao et al., 2024). Cadmium has been classified as a Group 1 carcinogen for humans by the International Agency for Research on Cancer (IARC) (IARC, 1993). Soil pollution caused by trace elements can be ecotoxic for plants (Domínguez et al., 2010). Research conducted on various plants has reported that Cd has a phytotoxic effect that can inhibit growth and potentially lead to plant death (Yi and Wang, 2017).

Although silicon (Si) is not an essential element, it has been shown to be closely related to the alleviation of metal toxicity in plants (Patrícia et al., 2008). The necessity of Si for the development of certain plant species, its benefits for growth and yield increase, its regulation of biochemical reactions, and its effects on increasing plant resistance to salt, toxic heavy metal, and pathogenic stress have been reported by many scientists (Horuz et al., 2017). Silicon also significantly increases drought tolerance in plants by strengthening both physiological and structural defenses (Franco-Navarro et al., 2025).The immobilization of toxic heavy metals occurs either through an increase in soil pH or the formation of silicate complexes. Additionally, the reduction of metal ions in the soil substrate, gene regulation related to metal translocation, chelation, and Si-stimulated enzymatic and non-enzymatic antioxidant systems are among the mechanisms of action in toxic heavy metal stress (Bhat et al., 2019). Mineral-Si is inherently alkaline, so its surface can easily carry large amounts of exchangeable cations and small amounts of exchangeable H^+^, which causes strong hydrolysis of exchangeable cations in the soil, resulting in high levels of NaOH in the soil solution and further increasing the soil pH (Ma et al., 2021). The resulting high soil pH has been shown to reduce the availability of cations such as Cd, Cr, Fe, Mn, and Zn (Wan et al., 2019).

By the mid-21st century, global food production needs to increase to meet the food and nutrition demand of nine billion people, despite challenges such as environmental pollution and water scarcity (Raza et al., 2025). Rajabi Dehnavi et al. (2020) referred to Sorghum (*Sorghum bicolor* L.), belonging to the Poaceae family, as the “King of Grains” and reported that it is cultivated in arid and semi-arid regions for feed, grain, bioenergy, and phytoremediation purposes. According to FAOSTAT, sorghum is among the top five cereal crops globally, surpassed only by wheat, rice, maize, and barley in production quantity (FAO, 2020). Sorghum species are valued for their rapid biomass accumulation and adaptability, making them useful for soil improvement and remediation. For example, Wang et al. (2023) showed that energy sorghum varieties can remove Cd from subtropical farmland soils, and Shafiei Darabi et al. (2016) demonstrated that *Sorghum bicolor* can absorb Cd, lead, and arsenic from irrigation wastewater. Singh et al. (2024) reported that sorghum is more tolerant to Cd than wheat, maize, and jack-bean. Exogenous Si application to plants has been shown to reduce Cd uptake and protect cellular organelles against Cd-induced damage through the regulation of metal transporter genes (Gheshlaghpour et al., 2021). Numerous studies have reported that Si mitigates the adverse effects of Cd in plants via various biochemical and physiological pathways under Cd stress (Song et al., 2021; Cai et al., 2022; Riaz et al., 2023).

The present study aims to evaluate the effects of Cd on sorghum plants and to examine the role of Si application in mitigating Cd accumulation. Specifically, Cd accumulation in different plant organs (roots, stems, leaves, and panicles) was analyzed, along with the influence of Si treatment on the morphological characteristics of sorghum under Cd stress.

## 2 Materials and methods

This research was carried out between June and September 2024 in the experimental garden of the School of Food, Agriculture and Livestock at Bingöl University. This is an open area, with sun all day long. Seeds belonging to the Master BMR sorghum variety were utilized as planting material in the soil obtained from Bingöl University’s Campus area. Characteristics of the soil are shown in **Table 1**. The pH and EC values of the soil were determined using a pH (Hanna HI 221) and EC meter (Jenco 3173) according to the method reported by Demiralay (1993) and Rhoades (1996), the total lime content was determined using a calcimeter (Scheibler type) (Allison and Moodie, 1965), and the organic matter content was determined using a modified Walkley-Black method (Nelson and Sommers, 1996). The plant-available Ca, Mg, and K ratios were measured using the 1 N ammonium acetate (NH_4_OAC, pH=7) method (Helmke and Sparks, 1996), and the plant-available P was measured using the 0.5 M NaHCO_3_ method reported by Olsen and Sommers (1982). Extractable Cd, Fe, and Zn were determined using the DTPA method (Lindsay and Norvell, 1978).

**Table 1:** Some characteristics of the soil used in the experiment.

pH	EC (µs cm^-1^)	Lime (CaCO_3_, %)		Organic matter (%)		K (mg kg^-1^)	P (mg kg^-1^)		Ca (mg kg^-1^)		Mg (mg kg^-1^)
6.88	190.3	1.96		0.38		354.5	21.23		7322.01		301.22

Extractable (mg kg^-1^)											
Cd		Si		Zn			Fe		Mn		
0.06		7.6		1.71			24.02		32.74		

**Figure 1:**
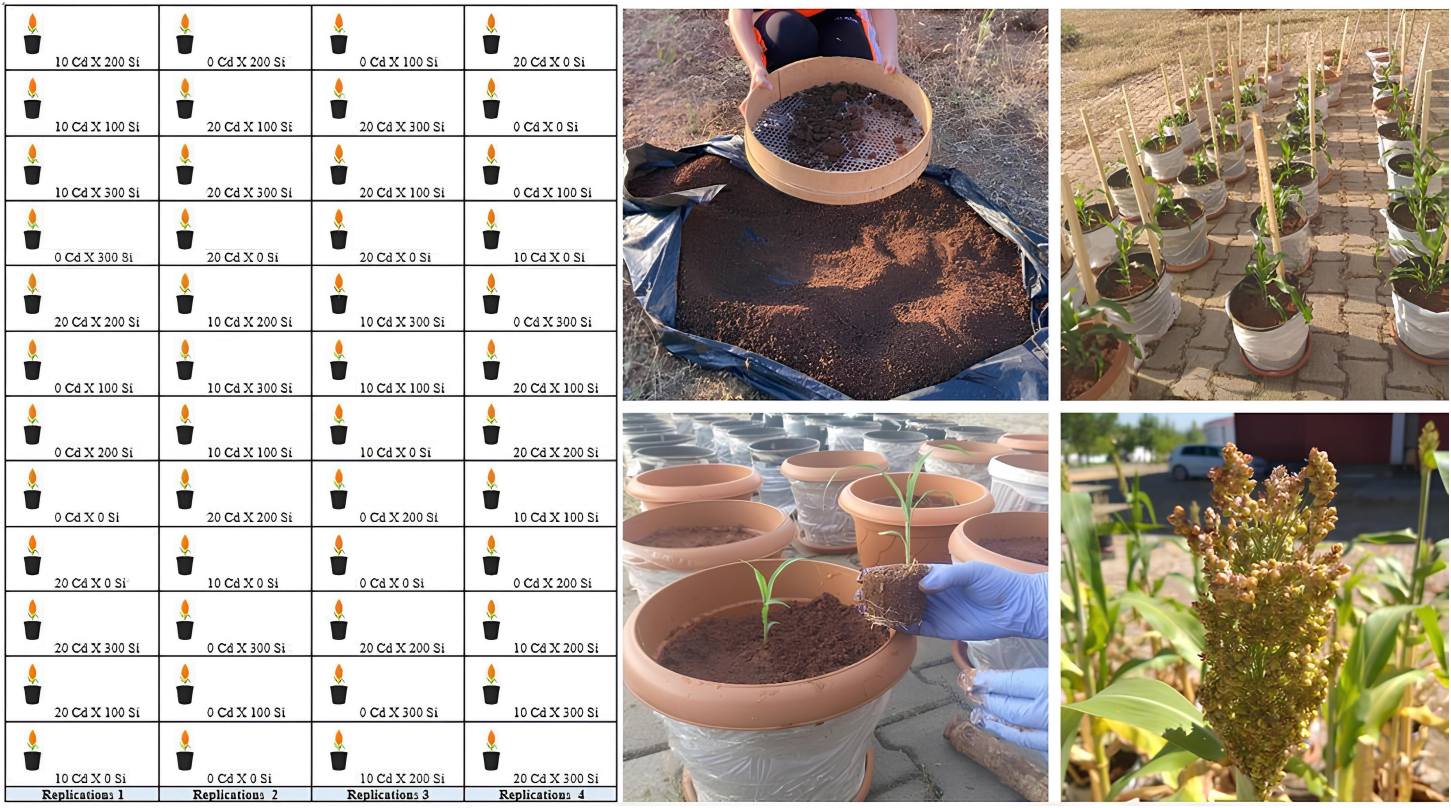
Views of the sorghum plant at different stages of growth.

The experiment was executed using a factorial design in completely randomized design (CRD). Cadmium concentrations were 0, 10, 20 mg kg^-1^, and Si concentrations were 0, 100, 200, 300 mg kg^-1^. Utilizing 48 pots for (3 Cd levels × 4 Si levels × 4 replications, dose combination: 0 Cd x 0 Si, 0 Cd x 100 Si, 0 Cd x 200 Si, 0 Cd x 300 Si, 10 Cd x 0 Si, 10 Cd x 100 Si, 10 Cd x 200 Si,10 Cd x 300 Si, 20 Cd x 0 Si, 20 Cd x 100 Si, 20 Cd x 200 Si, 20 Cd x 300 Si). After the combinations were formed, each combination was included only once in each replication. Dose combinations are shown in **Figure 1**. The doses used in previous studies were considered when determining the Si and Cd concentrations; An et al. (2022) used 20 mg kg^-1^ Cd and 200 mg kg^-1^ Si in maize, Gheshlaghpour et al. (2021) used 0, 25, and 50 mg kg^-1^ Cd with Si in basil plants, and Patrícia et al. (2008) used 0, 50, 100, 150, and 200 mg kg^-1^ Si and 20 mg kg^-1^ Cd doses in maize.

Soil was sieved to air-dry weight (4 mm), weighed at 10 kg, and transferred to pots. The pots were plastic production pots, 11.5 liters in volume, 25–30 cm in height, and 25–30 cm in width. Cadmium (3Cd3SO_4_ 8H_2_O) and Si (Na_2_SiO_3_) were commercially sourced. Before planting, 0, 10, and 20 mg kg^-1^ Cd were added, and watering was done only for a few days to ensure a homogeneous distribution of the added Cd in the soil (preventing Cd from leaching under the pot). A few days after the Cd application, 0, 100, 200, and 300 mg kg^-1^ of Si were added. A few days after the Si application, the recommended fertilization for sorghum plants, 20 kg da^-1^ N and 10 kg da^-1^ P_2_O_5_, was calculated and applied to the pots. Sorghum seedlings approximately 10 cm in size, previously grown in trays (after Si, Cd, and fertilization), were transferred to pots. A single plant was grown in each pot (Gökdere, 2025).

The sorghum seedlings transferred to pots were watered with tap water for 90 days without any water leakage from the pots. The harvesting process was carried out by cutting the above-ground organs (panicles, leaves, and stems) from the pots with pruning shears. The roots remaining in the pots were removed by turning the pots upside down one by one, picking out the roots, and washing them thoroughly with plenty of water. The combinations of the experimental design and the images of the plants at different growth stages are presented in **Figure 1**.

### 2.1 Determination of Cd, Fe, and Zn concentrations in panicles, stems, leaves, and roots

Plant samples were dried in an oven at 70 °C for 48 hours, then ground in a mill and weighed to 0.5 g. The samples were digested in a microwave total digester following the method reported by Miller (1998). The samples were transferred to the vessels of the CEM-MARS 6 microwave device and 10 ml of HNO_3_ (ISOLAB/≥65%) was added. The settings required for total digestion in the device (power: 1030–1800 W; power (%): 100; dissolution time: 20–25 minutes; pressure: 180 psi; temperature: 200 °C; hold time: 30 minutes) was performed. The samples were then diluted (50 ml) and filtered (watman filter paper) and made ready for reading on ICP-MS. After filtration and dilution, the Cd, Fe, and Zn concentrations were determined using an ICP-MS device (ICP-MS NexION^®^ 2000 ^C^ (PerkinElmer^®^Inc., USA). ICP-MS information, for Cd, R^2^: 0.999995, LOD: 0.06, LOQ: 0.15, for Fe, R^2^: 0.999990, LOD: 1,64, LOQ: 4.27, for Zn, R^2^: 0.991754, LOD: 0.25, LOQ: 0.72, replicates: 3 and recovery (^89^Y) 91.5%-126.1%.

Calculation process: Total in the plant (mg kg^-1^) Cd, Fe, Zn =It×F (I)

It = Reading value of the plant solution, F = dilution factor (multiplication factor)/sample weight

### 2.2 Statistical analysis

In the study conducted according to the factorial experimental design in randomized plots, two different analyses of variance (ANOVA) were performed on all data obtained from measurements and analyses using the “JMP 13.2.0” program (JMP, 2018). A two-way ANOVA was performed to compare the Si*Cd interaction among all applied doses, and statistically significant parameters were compared using the Tukey test (5%). To compare Si doses within a single Cd dose, a one-way ANOVA was performed, and significant parameters were compared using the LSD (least significant difference) test (5%).

## 3 Results

### 3.1 Cadmium accumulation in plant organs (mg kg^-1^)

Two-way ANOVA showed that the results regarding the effect of Cd*Si treatments at different doses on Cd concentrations accumulated in the plant’s organs were as significant as (*p* < 0.01), and the data are presented in **Table 2**.

**Table 2:** Effect of Cd*Si treatments at different doses on Cd concentrations and averages in plant organs.

Treatments		Root	Stem	Leaf	Panicle	Mean
Cd_0_*Si_0_		2.03 ± 0.03^d^	0.99 ± 0.20^e^	0.44 ± 0.03^f^	0.23 ± 0.03^e^	0.92^D^**
Cd_0_*Si_100_		1.56 ± 0.44^d^	0.91 ± 0.29^e^	0.36 ± 0.02^f^	0.22 ± 0.01^e^	**0.76^D^**
Cd_0_*Si_200_		1.36 ± 0.14^d^	0.82 ± 0.32^e^	0.28 ± 0.05^f^	0.24 ± 0.01^e^	**0.68^D^**
Cd_0_*Si_300_		1.34 ± 0.04^d^	0.83 ± 0.19^e^	0.28 ± 0.0^f^	0.26 ± 0.0^e^	**0.68^D^**
Cd_10_*Si_0_		16.61 ± 0.36^ab^	8.33 ± 0.32^cd^	3.82 ± 0.19^d^	4.09 ± 0.28^b^	**8.21^ABC^**
Cd_10_*Si_100_		15.69 ± 1.56^ab^	12.90 ± 1.10^b^	4.17 ± 0.23^cd^	2.40 ± 0.31^c^	**8.79^ABC^**
Cd_10_*Si_200_		10.26 ± 0.74^c^	11.94 ± 1.06^bc^	4.88 ± 0.12^ab^	1.33 ± 0.08^d^	**7.10^BC^**
Cd_10_*Si_300_		11.45 ± 1.55^c^	5.92 ± 0.08^d^	2.64 ± 0.38^e^	0.55 ± 0.08^e^	**5.14^C^**
Cd_20_*Si_0_		16.88 ± 1.66^ab^	9.53 ± 0.61^bcd^	4.03 ± 0.38^d^	3.71 ± 0.29^b^	**8.54^ABC^**
Cd_20_*Si_100_		15.31 ± 1.48^b^	11.71 ± 1.9^bc^	4.75 ± 0.28^abc^	5.41 ± 0.59^a^	**9.29^AB^**
Cd_20_*Si_200_		15.36 ± 0.61^b^	19.77 ± 3.23^a^	5.01 ± 0.14^a^	5.07 ± 0.28^a^	**11.30^A^**
Cd_20_*Si_300_		18.39 ± 0.67^a^	10.78 ± 2.94^bc^	4.22 ± 0.34^bcd^	2.00 ± 0.02^cd^	**8.85^ABC^**
Mean		10.52^A^**	**7.87^B^**	**2.91^C^**	**2.13^C^**	
Cd*Si	F-value	11.43	11.74	14.56	68.07	
	*p*-value	<,0001**	<,0001**	<,0001**	<,0001**	

**p<0.01 is significant. Capital letters indicate mean groups, lowercase letters indicate interaction groups.

Mean concentration values in plant organs as a result of treatments are shown in bold.

As a result of Cd*Si interaction, the lowest Cd accumulation in roots were observed in Cd_0_Si_0,100,200,300_ while the highest accumulation at Cd_20_Si_300_ treatments, in the stems the lowest values were recorded in Cd_0_Si_0,100,200,300_ and the highest in Cd_20_Si_200_ treatments, Similarly in leaves, the lowest Cd accumulation was found in Cd_0_Si_0, 100, 200, 300_ with the highest in Cd_20_Si_200_ treatments, for panicles the lowest accumulation occurred in Cd_0_Si_0,100,200,300_, Cd_10_Si_300_, whereas the highest was observed in Cd_20_Si_200_, Cd_20_Si_100_ treatments.

Among plant organs, the highest average Cd accumulation was determined in the roots of the plant (10.52 mg kg^-1^), and the lowest in the panicles (2.13 mg kg^-1^). The highest average Cd accumulation in all plant tissues (11.30 mg kg^-1^) was determined in the Cd_20_Si_200_ treatment.

**Figure 2:**
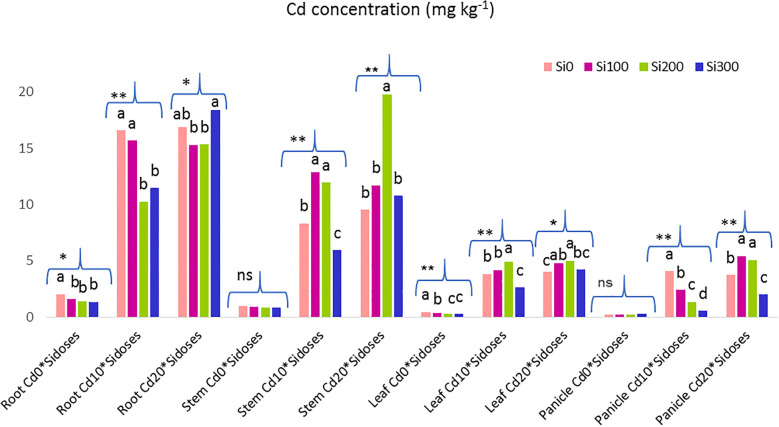
Graph showing the effect of Si doses on Cd content in plant organs at a single Cd dose. Lower case letters indicate significant difference between Si dose treatments at a single Cd dose. ns, non significant. **:p<0.01 and *:p<0.05 is significant.

The results of the one-way ANOVA conducted to evaluate the Si doses among themselves at a single Cd dose are shown in **Figure 2**, along with the Cd contents and significance levels of the plant organs.

Under Cd_10_ stress, Si applications caused a decrease in Cd concentration in roots and panicles, while under Cd_20_ stress, Si treatments caused an initial increase followed by a decrease in stem, leaf, and panicle Cd concentrations.

**Figure 3:**
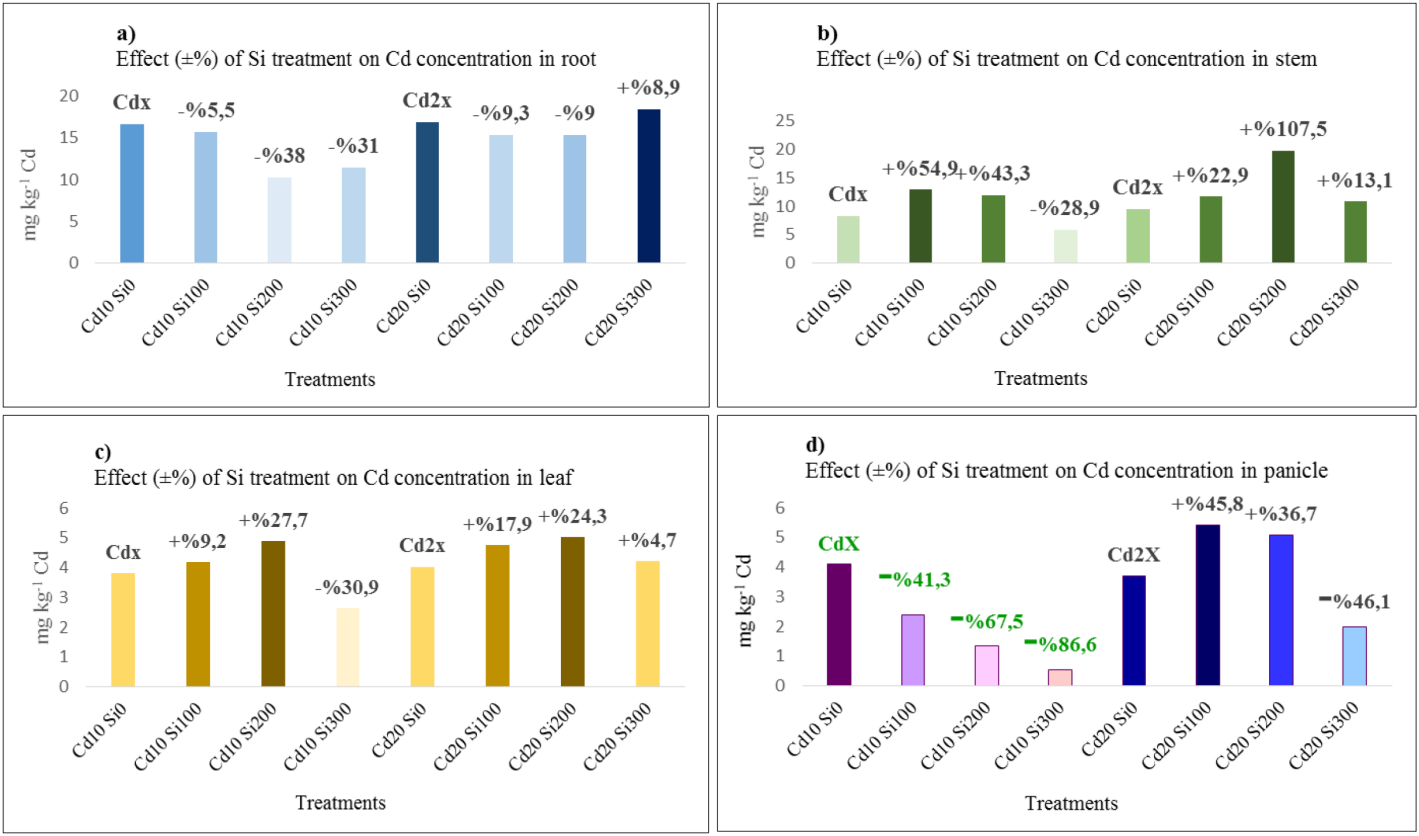
Percentage effect of Si treatment on Cd concentration in roots **(a)**, stems **(b)**, leaves **(c)**, and panicles **(d)**.

The percentage effect of Si treatments on Cd concentrations in roots, stems, leaves, and panicles is shown in **Figure 3**.

The most effective treatment for reducing Cd concentration in roots was Cd_10_Si_200_, while Cd_10_Si_300_ was the most significant treatment for reducing Cd accumulation in stems, leaves, and panicles. In the panicles, Cd content showed a consistent decrease parallel to the increase in Si doses, especially under Cd_10_ stress, and Cd content in the panicles was 86.6% lower in the Cd_10_Si_300_ treatment compared to the Cd_10_Si_0_ treatment.

### 3.2 Fe content of plant organs (mg kg^-1^)

Two-way ANOVA showed that Cd*Si applications at different doses had a significant effect (*p* < 0.01) on the Fe content of plant organs, and the data were presented in **Table 3**.

**Table 3:** Effect of Cd*Si treatments at different doses on Fe concentrations and averages in plant organs.

Treatments		Root	Stem	Leaf	Panicle	Mean
Cd_0_*Si_0_		4169.13 ± 279^a^	29.39 ± 1.84^c^	78.52 ± 0.84^a^	18.66 ± 3.25^de^	1073.93^A^**
Cd_0_*Si_100_		2650.50 ± 336^b^	22.47 ± 1.00^c^	64.75 ± 2.63^c^	14.20 ± 3.67^de^	**687.98^AB^**
Cd_0_*Si_200_		2634.42 ± 217^b^	23.69 ± 2.69^c^	45.12 ± 2.39^d^	10.38 ± 2.52^e^	**678.40^AB^**
Cd_0_*Si_300_		1552.09 ± 134^de^	26.48 ± 1.02^c^	85.65 ± 5.37^a^	19.69 ± 1.60^de^	**420.98^B^**
Cd_10_*Si_0_		1119.12 ± 143^def^	84.22 ± 8.78^a^	75.88 ± 5.43^ab^	29.91 ± 5.83^d^	**327.28^B^**
Cd_10_*Si_100_		1619.80 ± 111^cd^	96.00 ± 6.08^a^	59.14 ± 4.51^c^	20.28 ± 2.67^de^	**448.80^B^**
Cd_10_*Si_200_		2131.38 ± 145^bc^	95.67 ± 8.08^a^	67.23 ± 6.61^bc^	13.76 ± 3.26^de^	**577.01^AB^**
Cd_10_*Si_300_		1324.18 ± 192^def^	22.45 ± 2.20^c^	45.72 ± 4.18^d^	14.84 ± 2.91^de^	**351.80^B^**
Cd_20_*Si_0_		987.89 ± 187^f^	45.79 ± 3.98^b^	18.24 ± 2.52^f^	100.05 ± 11.1^a^	**287.99^B^**
Cd_20_*Si_100_		1023.59 ± 129^ef^	23.41 ± 1.23^c^	35.46 ± 2.71^de^	73.29 ± 1.24^b^	**288.94^B^**
Cd_20_*Si_200_		990.55 ± 118^ef^	49.66 ± 6.02^b^	31.90 ± 1.99^e^	53.02 ± 9.44^c^	**281.28^B^**
Cd_20_*Si_300_		1228.95 ± 106^def^	24.92 ± 2.44^c^	42.11 ± 2.59^de^	73.73 ± 7.81^b^	**342.43^B^**
Mean		1785.97^A^**	**45.35^B^**	**54.14^B^**	**36.82^B^**	
Cd*Si	F-value	44.97	55.62	56.25	7.78	
	*p*-value	<,0001^**^	<,0001^**^	<,0001^**^	0.0001^**^	

**p<0.01 is significant. Capital letters indicate mean groups, lowercase letters indicate interaction groups.

Mean concentration values in plant organs as a result of treatments are shown in bold.

As a result of Cd*Si interaction, Fe content was determined to be lowest in Cd_20_Si_0_ applications, highest in Cd_0_Si_0_ applications in roots, lowest in Cd_20_Si_0_ applications, highest in Cd_0_Si_0,300_ applications in leaves, lowest in Cd_0_Si_200_ applications, and highest in Cd_20_Si_0_ applications in panicles.

The average highest Fe content among plant organs (1785.97 mg kg^-1^) was determined in the roots of the plant, while the lowest Fe content (45.35, 54.14, and 36.82 mg kg^-1^) was determined in the stem, leaves, and panicles of the plant. The average highest Fe concentration in all plant tissues was determined to be 1073.93 mg kg^-1^ in the Cd_0_Si_0_ application.

**Figure 4:**
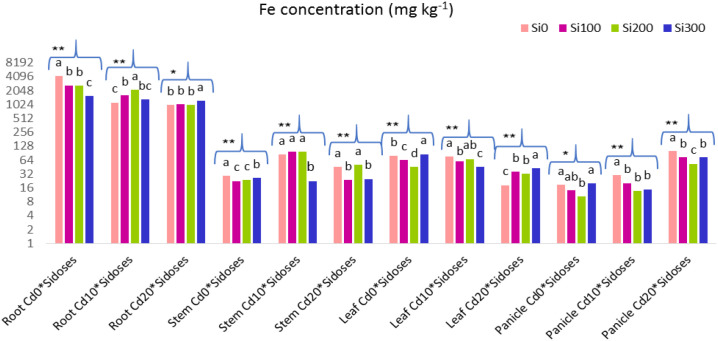
Graph showing the effect of Si doses on Fe content in plant organs at a single Cd dose. Lower case letters indicate significant difference between Si dose treatments at a single Cd dose. ns, non significant. **:p<0.01 and *:p<0.05 is significant.

The results of the one-way ANOVA conducted to evaluate the Si doses among themselves at a single Cd dose are shown in **Figure 4**, along with the Fe contents and significance levels of the plant organs.

Under Cd_10_ stress, Si treatments caused a decrease in Fe content in stems and panicles, while Cd_20_ stress caused an increase in roots and leaves.

### 3.3 Zn content of plant organs (mg kg^-1^)

Two-factor ANOVA showed that the effect of different doses of Cd*Si on Zn contents of plant organs was significant (*p* < 0.01), and the data were presented in **Table 4**.

**Table 4:** Effect of Cd*Si applications at different doses on Zn content and averages in plant organs.

Treatments		Root	Stem	Leaf	Panicle	Mean
Cd_0_*Si_0_		36.94 ± 1.73^a^	15.23 ± 2.75^ab^	7.67 ± 1.00^ab^	32.52 ± 0.73^b-e^	23.09^A^**
Cd_0_*Si_100_		26.73 ± 1.39^b^	13.02 ± 2.33^a-d^	3.62 ± 0.46^ef^	48.23 ± 4.11^abc^	**22.90^A^**
Cd_0_*Si_200_		16.66 ± 1.86^c^	12.60 ± 1.65^bcd^	6.16 ± 0.14^bcd^	49.16 ± 2.28^ab^	**21.15^AB^**
Cd_0_*Si_300_		10.31 ± 0.77^def^	15.18 ± 0.63^ab^	6.80 ± 0.90^a-d^	45.83 ± 1.56^a-d^	**19.53^ABC^**
Cd_10_*Si_0_		12.02 ± 1.25^de^	15.56 ± 1.66^ab^	4.45 ± 0.46^de^	56.02 ± 3.07^a^	**22.01^AB^**
Cd_10_*Si_100_		13.21 ± 1.14^cd^	19.17 ± 3.31^a^	5.21 ± 0.99^cde^	28.21 ± 6.48^de^	**16.45^ABC^**
Cd_10_*Si_200_		7.03 ± 0.52^fg^	13.73 ± 2.32^abc^	1.04 ± 0.21^g^	37.88 ± 3.13^a-e^	**14.92^A-D^**
Cd_10_*Si_300_		9.48 ± 1.22^ef^	8.51 ± 3.69^cde^	3.53 ± 0.44^ef^	31.00 ± 2.74^b-e^	**13.13^BCD^**
Cd_20_*Si_0_		5.03 ± 0.10^g^	15.18 ± 2.29^ab^	8.90 ± 0.68^a^	25.34 ± 4.12^e^	**13.61^BCD^**
Cd_20_*Si_100_		10.90 ± 1.35^de^	13.25 ± 4.06^abc^	8.22 ± 1.25^ab^	29.92 ± 6.48^cde^	**15.57^A-D^**
Cd_20_*Si_200_		9.22 ± 1.40^ef^	6.81 ± 1.62^de^	7.22 ± 1.42^abc^	21.93 ± 2.45^e^	**11.29^CD^**
Cd_20_*Si_300_		4.78 ± 0.62^g^	2.78 ± 0.38^e^	1.99 ± 0.12^fg^	18.74 ± 1.24^e^	**7.08^D^**
Mean		13.53^B^**	**12.59^B^**	**5.40^C^**	**35.40^A^**	
Cd*Si	F-value	**85.57**	**7.94**	**26.18**	**7.84**	
	*p*-value	<,0001^**^	0.0001^**^	<,0001^**^	<,0001^**^	

**p<0.01 is significant. Capital letters indicate mean groups, lowercase letters indicate interaction groups.

Mean concentration values in plant organs as a result of treatments are shown in bold.

As a result of Cd*Si interaction, the lowest Zn concentration in roots was observed in Cd_20_Si_0,300_ while the highest occurred in Cd_0_Si_0_ treatments. In stems, the lowest Zn concentration was recorded in Cd_20_Si_300_ and the highest in Cd_10_Si_100_ treatments. In leaves, the lowest value was found in Cd_10_Si_200_, whereas the highest was in Cd_20_Si_0_ treatments. In panicles, the lowest concentrations were observed in Cd_20_Si_0_,_200,300_ while the highest occurred in Cd_10_Si_0_ treatments. Among the plant organs, Zn content ranged from a minimum of 5.40 mg kg^-1^ in leaves to a maximum of 35.40 mg kg^-1^. Across the total plant tissue, the lowest Zn concentration (7.08 mg kg^-1^) was found in Cd_20_Si_300_, while the highest values, 23.09 and 22.90 mg kg^-1^, were recorded in Cd_0_Si_0,100_ treatments, respectively.

**Figure 5:**
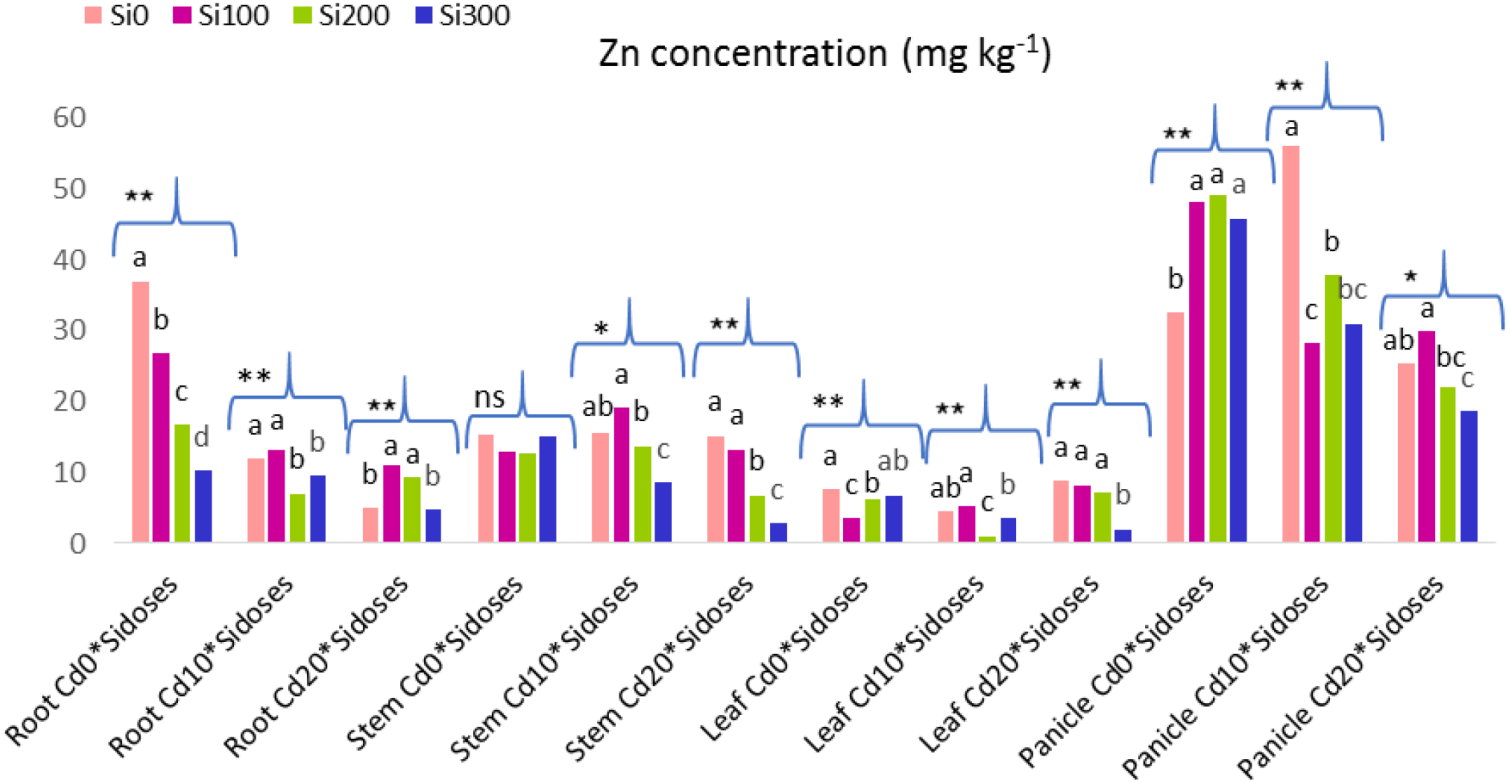
Graph showing the effect of Si doses on Zn content in plant organs at a single Cd dose. Lower case letters indicate significant difference between Si dose treatments at a single Cd dose. ns, non significant. **:p<0.01 and *:p<0.05 is significant.

The results of the one-way ANOVA conducted to evaluate the Si doses among themselves at a single Cd dose are shown in **Figure 5**, along with the Zn contents and significance levels of the plant organs.

In Cd_10_ stress, Si doses caused a decrease in Zn content in the root, while Cd_20_ stress caused a decrease in Zn content in the stem and leaves.

### 3.4 Plant morphological characteristics

Two-factor ANOVA showed that the effect of the different doses of Cd*Si treatment on some morphological traits of sorghum was not statistically significant, and the data are presented in **Table 5**.

**Table 5:** Effect of Cd*Si treatments at different doses on some morphological characteristics of sorghum.

Treatments		Plant height (cm)	Plant stem diameter (mm)	Panicle length (cm)	Dry root weight (g)	Total plant weight (g)
Cd_0_*Si_0_		111.33 ± 6.50	19.00 ± 2.20	15.33 ± 1.52	15.63 ± 1.05	70.43 ± 1.80
Cd_0_*Si_100_		103.67 ± 3.51	18.68 ± 1.22	14.67 ± 1.52	15.80 ± 1.30	73.83 ± 5.50
Cd_0_*Si_200_		105.33 ± 5.68	18.62 ± 0.37	13.00 ± 1	16.23 ± 2.51	82.10 ± 15.7
Cd_0_*Si_300_		102.66 ± 5.85	17.72 ± 0.51	13.66 ± 0.57	16.67 ± 1.52	86.47 ± 16.5
Cd_10_*Si_0_		108.00 ± 4.58	18.85 ± 0.36	14.00 ± 2	9.50 ± 2.10	70.90 ± 4.84
Cd_10_*Si_100_		108.00 ± 3.46	20.50 ± 1.32	14.66 ± 2.08	9.43 ± 2.15	80.47 ± 11.0
Cd_10_*Si_200_		108.67 ± 7.02	21.00 ± 1.22	13.68 ± 0.57	13.27 ± 2.06	88.30 ± 8.66
Cd_10_*Si_300_		110.33 ± 8.32	20.47 ± 0.22	13.31 ± 4.72	14.90 ± 1.99	93.93 ± 4.69
Cd_20_*Si_0_		106.00 ± 1.0	19.29 ± 1.41	14.00 ± 1	10.10 ± 1.92	65.93 ± 0.64
Cd_20_*Si_100_		105.00 ± 4.58	22.00 ± 1.27	15.33 ± 1.52	11.83 ± 3.38	73.90 ± 5.53
Cd_20_*Si_200_		103.00 ± 7.0	21.19 ± 0.61	15.00 ± 1.0	12.77 ± 4.46	88.20 ± 0.2
Cd_20_*Si_300_		101.33 ± 7.57	21.36 ± 0.68	13.67 ± 1.15	13.83 ± 0.85	95.20 ± 11.06
Cd*Si	F-value	0.56	1.74	1.05	0.77	0.36
	*p*-value	0.8454 ^ns^	0.1581 ^ns^	0.4191 ^ns^	0.6052 ^ns^	0.8979 ^ns^

ns, non significant.

Although not statistically significant, numerically, the highest plant height was in Cd_0_Si_0_, the thickest plant stem diameter in Cd_20_Si_100_, the longest panicle in Cd_0_Si_0_, the highest dry root weight in Cd_0_Si_300_, and the highest whole plant weight (biomass) in Cd_20_Si_300_ treatments were determined.

**Figure 6:**
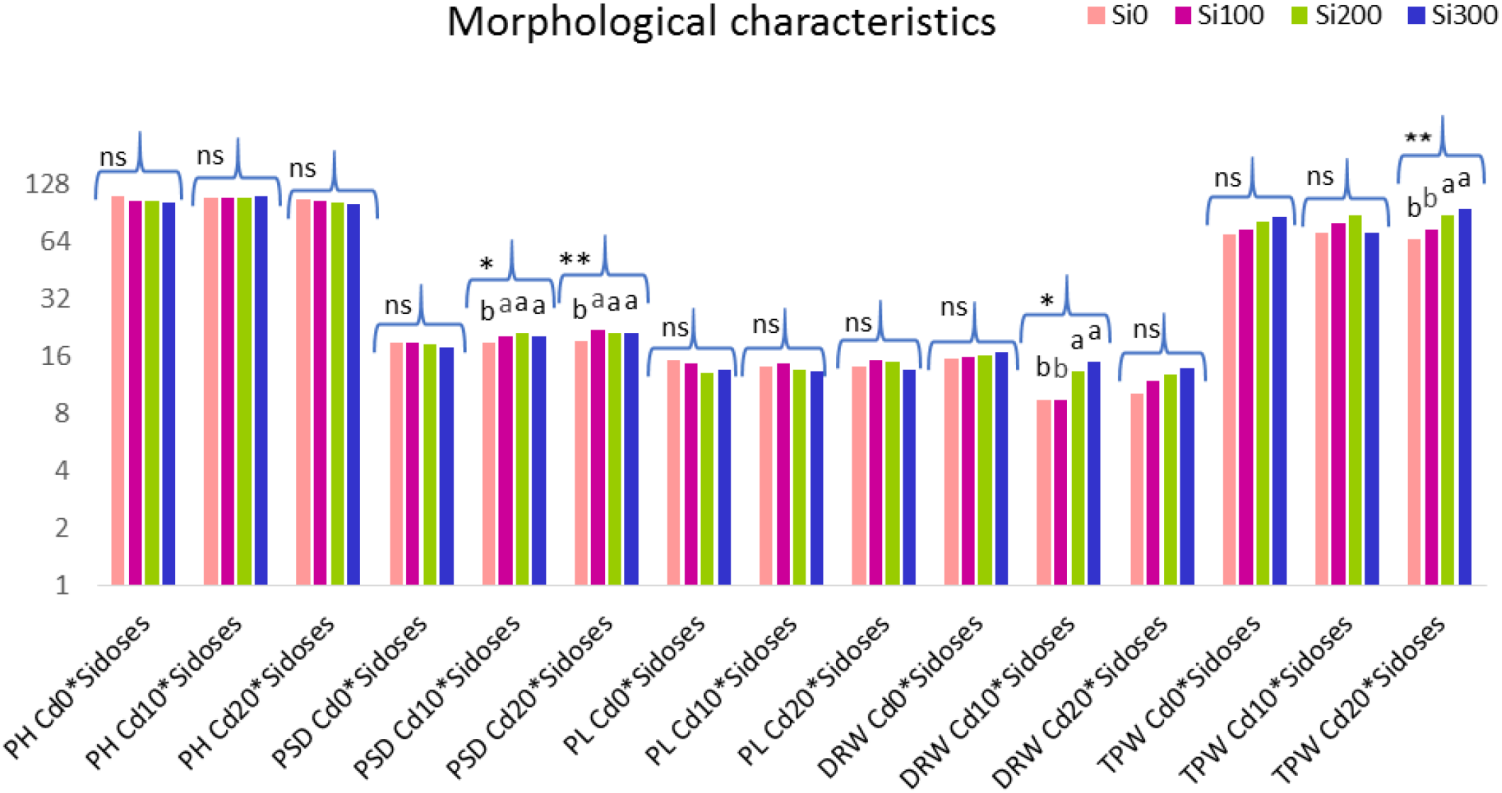
Graph of the effect of Si doses at a single Cd dose on some morphological characteristics of the plant. PH, plant height, PSD, plant stem diameter, PL, panicle length, DRW, dry root weight, TPW, total plant weight. Lower case letters indicate significant difference between Si dose treatments at a single Cd dose. ns, non significant. **:p<0.01 and *:p<0.05 is significant.

Some morphological traits of sorghum and their significance levels are shown in **Figure 6** as a result of one-factor ANOVA conducted for the evaluation of Si doses in a single Cd dose.

In Cd_10_ stress, Si doses caused an increase in plant stem diameter and dry root weight, while in Cd_20_ stress, Si doses caused an increase in plant stem diameter and whole plant weight.

## 4 Discussion

Cd accumulation in plant organs varied according to the Si dose and organ. Cd accumulation in the root decreased with Si treatments (except Cd_20_Si_300_), while in the stem, initially it increased and then decreased, and in the leaves, the effect of Si in reducing Cd concentration was observed in Cd_10_Si_300_ treatment. Cd concentration in panicles decreased in parallel with the increase in Si doses. Silicon treatment eliminated Cd accumulation in different organs differently, which is thought to be due to molecular and biochemical responses. Exogenous Si treatment was found to be effective in reducing Cd absorption and protecting organelles from Cd damage by regulating metal transporter genes (Gheshlaghpour et al., 2021). In rice plants, Si addition caused a decrease in the expression level of *OSLSI6* in leaf bases, but not in leaf sheaths (Yamaji et al., 2008). Jia et al. (2016) showed that the distribution of Cd entering sweet sorghum seedlings was not homogeneous in different tissues, with Cd localization mostly centralized in the stele of roots and distributed in the intercellular space of stems. The role of Si in reducing metal translocation into shoots is mediated by the accumulation of Si in the cell wall, which forms a barrier to the apoplastic pathway of metals, the formation of a physical barrier in the apoplast space reduces Cd transport rates, thus reducing the accumulation and distribution of Cd in the cytoplasm (Riaz et al., 2023). In the same Cd stress, different Si doses may cause different biochemical and molecular responses. Each organ may have different metabolic pathways and the capacity to remove metals. These differences may lead to differences in gene expression, metal transport, and stress responses. A high Si dose (Si 300) may have created more barriers to Cd in the cell, resulting in similar protection in different organs, whereas a low Si dose may have resulted in incomplete development of these barriers.

Some applied Si doses were observed to reduce Cd concentration in the root. Dresler et al. (2015) reported that Cd accumulation in the roots of maize seedlings decreased significantly with increasing concentrations of Si in the nutrient solution. An et al. (2022) reported that in Cd-stressed maize plants, Si treatment increased Cd concentration in the roots of the deep-rooted genotype but decreased it in the shallow-rooted genotype. Addition of Si to the nutrient solution has been reported to increase Cd accumulation in roots (Cunha and Nascimento, 2009; Song et al., 2011). The order among plant organs for Cd accumulation was leaf=panicle<stem<root. Among the reasons for the effect of Si on the accumulation of Cd in roots are that Si may prevent Cd transport to the stem and thus to the shoot by promoting the formation of a suberized barrier in the endodermal cell walls near the root tip (Vaculík et al., 2012), trapping Cd by binding to root cell walls and thus preventing cellular uptake of Cd (Liu et al., 2013), and Cd chelated by Si was retained in the vacuoles of plant root cells, leading to less translocation to the shoot (Zhang et al., 2018). In the variable movement of Cd concentration in the stem with Si treatment; Si increases the cation exchange capacity of the cell wall in some cases and Cd can bind to the cell wall more in the presence of Si than in its absence (Lukačová et al., 2013), Cd entry into the root occurs in two ways, apoplast and symplast (Song et al., 2017), and Si can interfere with these two transport pathways in various ways to prevent Cd accumulation in plants (Song et al., 2021).

The effect of Si in reducing Cd concentration in leaves was observed only in the Si_300_ treatment. Cadmium concentration decreased in panicles in parallel with the dose increase of Si in Cd_10_ stress, and Cd concentration in panicles decreased by 86.6% in Cd_10_Si_300_ treatment. Wei et al. (2018) reported that Si treatment activated the physiological reduction mechanism in the transport of toxic heavy metals from root to grain in paddy and Si treatment reduced Cd content in grains of paddy plants by 51.9%. Liu et al. (2020) reported that Cd concentration in grains of maize plants under Cd stress decreased by 66% with Si and NO treatments. An et al. (2022) reported that in two different Cd-treated maize genotypes, Si treatment reduced Cd concentration in grain by 14.4% (Zhongke11) and 21.4% (Shengrui999) compared to Cd-only treated plants. Rahman et al. (2023) reported that Cd content of grains was significantly reduced by 60.6% and 43.2% by Si treatment in maize plants under Cd stress (25 and 50 mg kg^-1^), respectively. Silicon symplastically inhibits the transport of Cd from shoots to grains in plants (Wu et al., 2019). In gramineous plants, elements taken up by the roots are not directly transported to the grains; the distribution of elements is redirected in the plant internodes. Therefore, element accumulation in panicles is thought to be an important step in the selective control of distribution in the internodes (Feng et al., 2011).

At 20 mg kg^-1^ Cd contamination, it is seen that Si doses applied to reduce Cd accumulation in plant organs were not successful enough. Snakin et al. (1997) and Kabata-Pendias (2011) stated in their studies that the increase in NaCl concentration in soils increases Cd uptake. In this experiment, Si was applied to the soils in the form of Na_2_SiO_3_. Considering the possibility of forming compounds with the existing CI^-^ anion in the soil with the addition of sodium (Na) to the soils, it can be thought that Si treatment at higher Cd doses could not alleviate the Cd uptake. Similarly, McLaughlin et al. (1994) reported that plants, especially sunflowers and potatoes, uptake significantly more Cd from salt-affected soils.

Among the plant organs, the highest Fe content was measured in roots; however, different doses of Si treatments decreased Fe concentration in panicles, leaves, stems, and roots compared to Si_0_. Pavlovic et al. (2021) suggested that Si treatment reduced Fe uptake and transport to the above-ground parts in paddy. Recent research on rice has reported that the addition of Si to the growth medium causes more Fe plaque formation, thereby reducing Fe uptake and activating root Fe deficiency responses even in optimal Fe availability. Fe concentration in roots and leaves decreased with increasing Cd doses. The presence of Cd in plants inhibits not only Fe absorption but also Fe translocation from the subsoil to the above-ground parts of the plant (Xu et al., 2015). In panicles, Fe concentration increased without being affected by Cd dose increase, and this is thought to be due to the fact that Cd accumulation in plant organs is the lowest in panicles; since Cd is less carried to panicles, Fe movement to panicles may not have been inhibited.

Cadmium and Si treatments generally resulted in reduced Zn concentrations in panicles, leaves, stems, and roots. Silicon has been reported to inhibit Zn uptake in several plant species, such as rice and maize (Dresler et al., 2015). Cadmium and various microelements, including Mn, Zn, Cu, and Fe, interact antagonistically and synergistically in their uptake and transport into the plant (Shahzad et al., 2025). Mostly, however, the interaction between Cd and Zn is antagonistic; Zn and Cd were found to be antagonistic in lettuce, spinach, wheat, chicory, and maize plants, Cd reduced plant uptake of Zn, and the reverse was less common (Smilde et al., 1992). Many studies have reported that Si reduces the bioavailability of Zn in soil by breaking it down into more stable fractions such as organic matter and crystalline Fe-oxides (da Cunha et al., 2008). Greger et al. (2018) reported that Si reduced net Zn accumulation in several plant species (maize, lettuce, wheat, carrot, and pea), even when there was sufficient Zn supply, although root Zn concentration increased. Therefore, the researchers concluded that the binding of Zn to Si in the roots prevents the transport of zinc to the shoot (Pavlovic et al., 2021). Nutrient exchange under Cd stress depends on plant species, genotypes, and plant organs (Rasafi et al., 2022).

Cadmium accumulation in plant tissues increased with increasing Cd concentrations compared to control plants, but the morphological characteristics of sorghum were not statistically affected. It has been shown that sorghum plants are highly resistant to metal pollution and can reach high biomass even in the presence of toxic metals (Soudek et al., 2014). Jia et al. (2016) reported that sweet sorghum maintained almost normal growth when exposed to 10 μM Cd for 30 days. The use of sorghum in phytoremediation is more suitable for moderate or low Cd pollution conditions (≤ 30 mg kg^-1^). In areas contaminated with low levels of Cd, the high biomass of sweet sorghums contributed to Cd uptake (Xiao et al., 2021a). Moreover, Cd concentrations in agricultural soils were lower than in pot experiments, but most sweet sorghum cultivars were able to grow normally (Xiao et al., 2021b). Padmapriya et al. (2016) investigated the performance of millet, mustard, sorghum, lentil, and squash in toxic heavy metal-contaminated soils and found that sorghum showed no significant change in biomass and biochemical parameters compared to the control. It could be observed that some Cd doses had a positive effect on plant stem diameter development. In some studies, it was reported that Cd treatment had a positive effect on plant growth, contrary to expectations. Cadmium treatment increased tobacco biomass (Liu et al., 2011). Zhang et al. (2002) administered Cd to wheat plants at varying doses during the seedling period and reported that low doses of Cd positively affected growth and dry matter accumulation. Carvalho et al. (2018) reported that the fruit diameter of tomato grown in Cd -contaminated soil increased due to exogenous Cd treatment. Dry root and whole plant weight increased at some Si doses. Cai et al. (2020) observed positive effects of Si applied at different growth stages on biomass and yield of paddy plants. Cunha and Nascimento (2009) reported that Si treatment to maize grown in Cd-treated soils led to an increase in plant biomass. Exogenous Si treatment increased the biomass of roots, stems, and leaves of tobacco plants under Cd stress (Yingang et al., 2018).

## 5 Conclusion

When the role of Si treatment in reducing Cd exposure in sorghum was examined, it was observed that at 20 mg kg^-1^ Cd contamination, Si applications were not effective enough to reduce Cd accumulation in plant organs. Cd_10_Si_300_ treatments reduced Cd concentrations by 86.6% in panicles, 30.9% in leaves, and 28.9% in stems, while Cd_10_Si_200_ applications reduced Cd concentrations in roots by 38% at most. In Cd translocation to panicles, it was observed that the treatment of 300 mg kg^-1^ Si had a strong potential to inhibit translocation in the presence of about 10 mg kg^-1^ Cd. In the soils with similar properties and Cd contamination used in this study, the treatment of 300 mg kg^-1^ Si is an important concentration to overcome Cd translocation.

## Data Availability

The original contributions presented in the study are included in the article/supplementary material. Further inquiries can be directed to the corresponding author.
